# Detection, prevalence, and transmission of avian hematozoa in waterfowl at the Arctic/sub-Arctic interface: co-infections, viral interactions, and sources of variation

**DOI:** 10.1186/s13071-016-1666-3

**Published:** 2016-07-07

**Authors:** Brandt W. Meixell, Todd W. Arnold, Mark S. Lindberg, Matthew M. Smith, Jonathan A. Runstadler, Andrew M. Ramey

**Affiliations:** Department of Fisheries, Wildlife, and Conservation Biology, University of Minnesota, St. Paul, MN 55108 USA; U.S. Geological Survey, Alaska Science Center, Anchorage, AK 99508 USA; Institute of Arctic Biology and Department of Biology and Wildlife, University of Alaska Fairbanks, Fairbanks, AK 99775 USA; Department of Biological Engineering and Division of Comparative Medicine, Massachusetts Institute of Technology, Cambridge, MA 02139 USA

**Keywords:** Hematozoa, Blood parasites, Co-infection, Occupancy models, Detection probability, *Haemoproteus*, *Leucocytozoon*, *Plasmodium*, Influenza A Virus, Waterfowl

## Abstract

**Background:**

The epidemiology of avian hematozoa at high latitudes is still not well understood, particularly in sub-Arctic and Arctic habitats, where information is limited regarding seasonality and range of transmission, co-infection dynamics with parasitic and viral agents, and possible fitness consequences of infection. Such information is important as climate warming may lead to northward expansion of hematozoa with unknown consequences to northern-breeding avian taxa, particularly populations that may be previously unexposed to blood parasites.

**Methods:**

We used molecular methods to screen blood samples and cloacal/oropharyngeal swabs collected from 1347 ducks of five species during May-August 2010, in interior Alaska, for the presence of hematozoa, Influenza A Virus (IAV), and IAV antibodies. Using models to account for imperfect detection of parasites, we estimated seasonal variation in prevalence of three parasite genera (*Haemoproteus*, *Plasmodium*, *Leucocytozoon*) and investigated how co-infection with parasites and viruses were related to the probability of infection.

**Results:**

We detected parasites from each hematozoan genus in adult and juvenile ducks of all species sampled. Seasonal patterns in detection and prevalence varied by parasite genus and species, age, and sex of duck hosts. The probabilities of infection for *Haemoproteus* and *Leucocytozoon* parasites were strongly positively correlated, but hematozoa infection was not correlated with IAV infection or serostatus. The probability of *Haemoproteus* infection was negatively related to body condition in juvenile ducks; relationships between *Leucocytozoon* infection and body condition varied among host species.

**Conclusions:**

We present prevalence estimates for *Haemoproteus*, *Leucocytozoon*, and *Plasmodium* infections in waterfowl at the interface of the sub-Arctic and Arctic and provide evidence for local transmission of all three parasite genera. Variation in prevalence and molecular detection of hematozoa parasites in wild ducks is influenced by seasonal timing and a number of host traits. A positive correlation in co-infection of *Leucocytozoon* and *Haemoproteus* suggests that infection probability by parasites in one or both genera is enhanced by infection with the other, or that encounter rates of hosts and genus-specific vectors are correlated. Using size-adjusted mass as an index of host condition, we did not find evidence for strong deleterious consequences of hematozoa infection in wild ducks.

**Electronic supplementary material:**

The online version of this article (doi:10.1186/s13071-016-1666-3) contains supplementary material, which is available to authorized users.

## Background

Protozoan blood parasites of the order Haemosporidia, or hematozoa, are transmitted by arthropod vectors and infect a broad diversity of avian taxa. The three most widely studied genera of hematozoa, *Leucocytozoon*, *Haemoproteus*, and *Plasmodium*, are putatively transmitted by the genus-specific dipteran vectors: black flies (Simuliidae), biting midges (Ceratopogonidae), and mosquitoes (Culicidae), respectively [1]. Endemic lineages of blood parasites circulating in wild bird populations may be associated with low virulence as evidence of direct mortality is rare, presumably due to long-term, host-pathogen evolutionary associations [2]. However, exposure of naïve hosts to hematozoa parasites may cause mortality and morbidity. For instance, the introduction of *Plasmodium* parasites to endemic Hawaiian avian taxa is thought to be responsible for reductions in abundance of native bird populations [3–5], and hematozoa infection can cause high rates of mortality in domestic birds (reviewed by [2]). Furthermore, species-specific variation in pathogenic outcomes resulting from experimental inoculation, ranging from 100 % mortality to resistance, suggests that susceptibility and virulence are influenced by host resistance developed through co-adaptation [6–9].

Environmental conditions influence the distribution and transmission of avian blood parasites [10, 11], and in North America, the abundance and diversity of avian hematozoa appears to vary across ecoregions [12]. Although *Leucocytozoon*, *Haemoproteus*, and *Plasmodium* parasites are known to infect birds in northern regions of North America, prevalence of these parasites vary. *Leucocytozoon* parasites appear to be the most common and widely distributed avian hematozoa at high latitudes, having been identified in juvenile or non-migratory resident birds as far north as the Arctic Coastal Plain [13–17], which is indicative of local transmission and completion of the parasite life-cycle. Although generally identified at lower prevalence, *Haemoproteus* and *Plasmodium* parasites are also transmitted among birds in temperate and sub-arctic regions [14, 17–20], but only sporadic infections of these genera have been detected north of the Arctic Circle, and to date, there is no direct evidence of *Plasmodium* transmission among birds in the North American Arctic [13, 21, 22]. Therefore, the geographic leading edge of *Plasmodium* and *Haemoproteus* transmission in North America appears to be at the interface of the Arctic and sub-Arctic.

Changing climatic conditions in arctic regions, such as accelerated warming [23], may increasingly favor northward range expansion of hematozoa [24, 25]. In North America, hematozoa transmission occurs primarily during summer months on the breeding grounds [1, 26], presenting the potential for parasite range expansion into previously unexposed bird populations that breed in arctic regions [17, 21]. Climatic changes may also promote increases in parasite transmission and prevalence in areas where parasites are endemic. These concerns highlight the need for information on prevalence, transmission, and distribution of blood parasites [27], especially at the presumed leading edge of their geographic range where infections may pose fitness consequences on naïve hosts.

Additionally, to understand dynamics of infection, it is important to consider sources of variation from heterogeneity in the distribution, abundance, and behavior of vectors and hosts [19, 28, 29]; as a result of genetically based disparities in host immunocompetence [6, 30, 31]; and on account of co-infections. Previous research [9, 32–34] suggests that infection by a given hematozoa lineage may increase or decrease susceptibility to infection by another, and that concomitant interactions among hematozoa lineages influence both parasite persistence and pathogenic outcome. Hematozoa infections have also been shown to interact in synergistic and antagonistic relationships with viruses and other pathogens in birds and mammals [3, 20, 35–37]. As such, thorough understanding of the epidemiology of hematozoa in wild birds requires not only knowledge of within- and among-host variation in transmission and prevalence of individual infections, but must also take into consideration the occurrence of multiple infections and the interactions between and among infectious agents within hosts. One viral agent that may potentially influence hematozoa infection in high-latitude waterfowl populations is Influenza A Virus (IAV).

IAV commonly infects waterfowl and is characterized by seasonal peaks of infection, especially among immunologically naïve juveniles, during late summer and autumn [38]. IAV strains common to wild waterfowl are generally not associated with mortality or morbidity; however, some evidence suggests that low pathogenic IAV infection may be associated with reductions in body weight, delayed migration, and pathological lesions in waterfowl hosts (reviewed by [39]). Similar to IAV, hematozoa infection has been linked to body condition in wild ducks [40], and fitness consequences associated with infection have been reported in a variety of avian taxa (e.g. [41–44]), although natural infections may pose minimal deleterious effects to wild waterfowl [2, 40, 45, 46]. As both IAV and hematozoa can infect waterfowl at relatively high rates and elicit immune responses, and such infections have been associated with sub-lethal effects in other avian taxa, investigation of co-infection and consequences of infection in waterfowl are warranted. Such information would be useful for further understanding of interactions among co-infecting parasitic and viral agents, and for predicting potential outcomes should range expansion or changes in population-level prevalence of hematozoa occur.

In this study, we investigated seasonal patterns and sources of variation in prevalence of *Leucocytozoon*, *Haemoproteus*, and *Plasmodium* parasites among five species of ducks breeding at the Arctic/sub-Arctic interface in the boreal forest region of interior Alaska. Our goal was to better understand within- and among-species variation in prevalence, identify specific factors related to the probability of infection and co-infection, assess evidence for local transmission at the northern periphery of the sub-Arctic, and to better understand potential fitness consequences of infection and co-infection. Specifically, we used molecular detection of hematozoa and an occupancy modeling approach, which accounts for imperfect detection rates, to: (i) estimate genus-specific hematozoa prevalence relative to month, species, age, and sex, (ii) evaluate co-infection dynamics among and between hematozoa genera and IAV, and (iii) assess relationships between size-adjusted body mass and single and multiple infections.

## Methods

### Study area

**Fig. 1:**
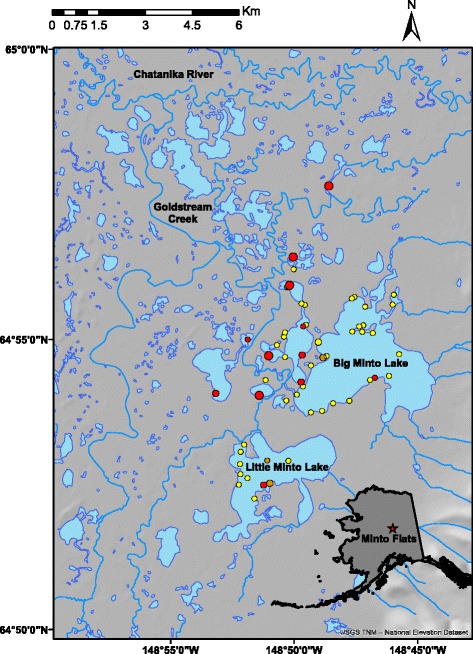
Study area and capture locations within Minto Flats State Game Refuge, Alaska, USA. Circles depict capture locations relative to monthly sampling intervals (*yellow* = 17–27 May, *orange* = 10–19 June, *red* = 28 July – 20 August) and number of birds sampled (small = 1–25, medium = 26–100, large = 100–200)

We conducted our study at Minto Flats State Game Refuge (Minto Flats; 64°53′N, 148°46′W) in the boreal forest region of interior Alaska, approximately 50 km northwest of Fairbanks and 180 km south of the Arctic Circle (Fig. 1). Our efforts were focused within approximately 100 km^2^ consisting of a series of interconnected lakes, seasonal wetlands, and streams surrounded by a mixture of grass, shrub, and forested uplands. Minto Flats supports high densities of breeding, molting, and migrating waterfowl and other species of waterbirds and landbirds [47]. Petrula [47] and Walker et al. [48] provide detailed descriptions of the Minto Flats study area.

### Sample collection

We captured and sampled 1347 ducks of 5 species between 17 May and 20 August 2010: American green-winged teal (hereafter: green-winged teal; *Anas crecca*, *n* = 228), American wigeon (*Anas americana*, *n* = 88), mallard (*Anas platyrhynchos*, *n* = 481), northern pintail (*Anas acuta*, *n* = 490), and lesser scaup (*Athya affinis*, *n* = 60) (Table 1). Sampling was conducted over three distinct periods: 17–27 May (May), 10–19 June (June), and 28 July – 20 August (August) and ducks were sampled temporally throughout each interval. Capture techniques varied depending on the timing of the season and included decoy traps (May) [49], rocket nets (June, August) [50], and baited swim-in traps (August) [51]. Upon capture, up to 2 cc of blood was obtained from the jugular vein of each bird; approximately 1 cc of whole blood for screening of hematozoa parasites was transferred immediately to cryovials; the remaining blood was transferred to serum separator tubes, allowed to clot for ≥ 2 h, centrifuged at 3000 rpm for up to 10 min, and then serum for screening of IAV antibodies was transferred to cryovials. Cloacal and oropharyngeal swabs to test for IAV infection were obtained from each bird and stored individually in cryovials containing viral transport media (M4RT, Remel, KS). All samples were kept cool for up to 3 h following collection, then frozen in liquid nitrogen vapor shippers at -150 °C, and once transported from the field site, maintained at -80 °C until analysis. We categorized bird age as hatch year (juvenile) or after hatch year (adult) using cloacal and plumage characteristics [52, 53]. Birds were weighed to the nearest 1.0 g using a digital scale and the lengths of total tarsus and head were measured to the nearest 0.1 mm using dial calipers [54].

**Table 1:** Sample sizes, number of positives, and apparent prevalence (in parentheses) of hematozoa, Influenza A Virus (IAV), and IAV serostatus (Ser)

Species	Age	Sex	Sampling month(s)	*n*	Leuc	Haem	Plas	AIV	Ser	Leuc & Haem	Leuc & Plas	Leuc & AIV	Haem & AIV	Plas & AIV	Leuc & Ser	Haem & Ser	Plas & Ser
MALL	Ad	M	May, Jun, Aug	137	91 (66)	71 (52)	4 (3)	27 (20)	56 (41)	51 (37)	4 (3)	19 (14)	11 (8)	2 (1)	40 (29)	30 (22)	2 (1)
		F	May, Jun, Aug	115	57 (50)	30 (26)	9 (8)	15 (13)	41 (36)	20 (17)	4 (3)	8 (7)	4 (3)	0 (0)	18 (16)	12 (10)	3 (3)
	Juv	M	Aug	118	13 (11)	6 (5)	18 (15)	21 (18)	10 (8)	1 (1)	2 (2)	3 (3)	4 (3)	5 (4)	1 (1)	1 (1)	3 (3)
		F	Aug	111	13 (12)	5 (5)	9 (8)	16 (14)	14 (13)	1 (1)	1 (1)	2 (2)	0 (0)	0 (0)	1 (1)	3 (3)	0 (0)
NOPI	Ad	M	May, Jun, Aug	105	47 (45)	20 (19)	5 (5)	3 (3)	35 (33)	13 (12)	3 (3)	1 (1)	0 (0)	0 (0)	19 (18)	9 (9)	2 (2)
		F	May, Jun, Aug	142	82 (58)	47 (33)	32 (23)	9 (6)	46 (32)	30 (21)	16 (11)	4 (3)	3 (2)	2 (1)	31 (22)	17 (12)	10 (7)
	Juv	M	Aug	119	47 (39)	8 (7)	35 (29)	8 (7)	11 (9)	5 (4)	16 (13)	2 (2)	0 (0)	2 (2)	7 (6)	1 (1)	5 (4)
		F	Aug	124	47 (38)	8 (6)	42 (34)	10 (8)	11 (9)	4 (3)	20 (16)	4 (3)	2 (2)	4 (3)	2 (2)	0 (0)	3 (2)
AGWT	Ad	M	Aug	54	29 (54)	13 (24)	1 (2)	3 (6)	22 (41)	9 (17)	1 (2)	2 (4)	0 (0)	0 (0)	12 (22)	5 (9)	0 (0)
		F	Aug	29	19 (66)	10 (34)	3 (10)	2 (7)	8 (28)	9 (31)	2 (7)	1 (3)	1 (3)	0 (0)	4 (14)	1 (3)	1 (3)
	Juv	M	Aug	101	29 (29)	6 (6)	9 (9)	10 (10)	10 (10)	4 (4)	2 (2)	4 (4)	1 (1)	0 (0)	2 (2)	0 (0)	1 (1)
		F	Aug	44	7 (16)	1 (2)	3 (7)	5 (11)	7 (16)	0 (0)	0 (0)	2 (5)	0 (0)	1 (2)	0 (0)	0 (0)	2 (5)
AMWI	Ad	M	May, Jun	76	66 (87)	39 (51)	6 (8)	2 (3)	5 (7)	36 (47)	6 (8)	1 (1)	1 (1)	0 (0)	5 (7)	2 (3)	2 (3)
		F	May, Jun	12	8 (67)	2 (17)	0 (0)	0 (0)	1 (8)	2 (17)	0 (0)	0 (0)	0 (0)	0 (0)	1 (8)	0 (0)	0 (0)
LESC	Ad	M	May	48	31 (65)	13 (27)	3 (6)	0 (0)	12 (25)	10 (21)	1 (2)	0 (0)	0 (0)	0 (0)	9 (19)	5 (10)	0 (0)
		F	May	12	2 (17)	3 (25)	0 (0)	0 (0)	3 (25)	1 (8)	0 (0)	0 (0)	0 (0)	0 (0)	1 (8)	1 (8)	0 (0)
Total				1347	588 (44)	282 (21)	179 (13)	131 (10)	292 (22)	196 (15)	78 (6)	53 (4)	27 (2)	16 (1)	153 (11)	87 (6)	34 (3)

Hematozoa genera were abbreviated (Leuc *Leucocytozoon*, Haem *Haemoproteus*, Plas *Plasmodium*) and the “&” indicates co-infections with multiple hematozoa genera and/or IAV and Serostatus. Sampling month refers to the intervals when birds were sampled (May = 17 May – 27 May; Jun = 10 June – 19 June; Aug = 28 July – 20 August). Age was classified as adult (Ad) or juvenile (Juv). Species *abbreviations*: *MALL* mallard, *NOPI* northern pintail, *AGWT* American green-winged teal, *AMWI* American wigeon, *LESC* lesser scaup

### Laboratory analysis

Blood parasites were detected using molecular methods as reported by Ramey et al. [55]. DNA was extracted from samples using the DNeasy Blood and Tissue Kit (Qiagen, Valencia, CA) and screened for hematozoa using the nested PCR protocol as described by Hellgren et al. [56]. A minimum of one negative control was incorporated into PCR reactions for every 24 wells. Reactions were conducted in eight-well strip tubes with individual caps that remained closed, except while loading template and reagents, to prevent cross contamination. Amplicons were visualized on 0.8 % agarose gels stained with Gel Red Nucleic Acid Gel Stain 10,000× in DMSO (Biotium, Hayward, CA). All samples were screened twice for hematozoa by nested PCR.

A 479-base pair (bp) cytochrome *b* (cyt *b*) mitochondrial DNA (mtDNA) target fragment was sequenced for all samples that had amplicons present on agarose gels. PCR products were treated with ExoSap-IT (USB Inc., Cleveland, OH) per the manufacturer’s instructions and not otherwise purified prior to sequencing. Sequencing was performed with identical primers used for PCR and with BigDye Terminator version 3.1 mix (Applied Biosystems, Foster City, CA) on an Applied Biosystems 3730xl automated DNA sequencer (Applied Biosystems, Foster City, CA). Sequence data were cleaned and edited using Sequencher version 5.1 (Gene Codes Corp., Ann Arbor, MI). Infections were assigned to genera (*Leucocytozoon*, *Haemoproteus*, or *Plasmodium*) using the nucleotide BLAST function available through the National Center for Biotechnology Information (NCBI). Assignment was based on the top NCBI BLAST result with a minimum identity score of at least 90 %. A sample was considered as positive for blood parasite infection in any given PCR run if it resulted in double-stranded, target mtDNA that was verified through genetic sequencing and successfully assigned to one of three parasite genera using BLAST. Genetic characterization of resultant sequences were previously summarized [57]. Single-stranded sequences or products that could not be assigned via BLAST (*n* = 20) were considered as negative to reduce the possible occurrence of false positives. To confirm the competency of extracted DNA, a 695-bp fragment of the mtDNA cytochrome  *c* oxidase I (COI) gene of waterfowl hosts was amplified using PCR protocols described by Kerr et al. [58].

Avian influenza virus was detected in swab samples using molecular methods as described by Runstadler et al. [59]. We extracted viral RNA using the MagMax-96 Viral Isolation Kit (Ambion Inc., Austin, TX) and screened RNA for IAV using a two-step real-time Reverse Transcriptase - Polymerase Chain Reaction (rRT-PCR) targeting the matrix gene [59]. The rRT-PCR assays were run on an ABI 7500 real-time PCR System (Applied Biosystems, Foster City, CA). Swab samples were considered positive for IAV RNA if Ct values were ≤ 45. We screened serum samples for the presence of IAV antibodies using a commercial blocking enzyme-linked immunosorbent assay (bELISA, FlockChek AI MultiS-Screen Antibody Test Kit, IDEXX Laboratories, Westbrook, ME) according to the manufacturer’s instructions [60].

### Statistical analyses

Previous studies using molecular identification of hematozoa mtDNA from blood samples have demonstrated high, but variable test sensitivity, usually within the range of 0.75 to 0.95 for single PCR runs [13, 14, 55, 61]. When unaccounted for, the occurrence of false negatives leads to underestimates of infection prevalence [62], so we used occupancy modeling to simultaneously estimate the probability of infection by each genera of hematozoa (Ψ_g_; prevalence), and the probability of molecular detection of each genera of hematozoa, given infection (*p*_g_). In this framework, identification of parasite mtDNA for each of the two nested PCR reactions from each bird was used to construct encounter occasions where the presence of mtDNA in a sub-sample of blood was indicated by a “1” whereas absence was indicated by a “0” and these data were used to estimate *p*_g_ and Ψ_g_ using maximum likelihood approaches [63]. If parasites are always detected in both samples (i.e. encounter history is always 1,1), then the data suggest an absence of false negatives, with observed prevalence equal to true prevalence. However, if there are instances of partial detections (encounter histories of 0,1 or 1,0), then these data provide information on detection probability (*p*), which can be used to estimate the proportion of samples where parasite mtDNA was detected in neither PCR run (i.e. some encounter histories of 0,0 represent infected birds that went undetected in both samples). We compiled encounter histories relative to each of the three parasite genera independently to allow for genus-specific estimation of hematozoa prevalence and detection. Whereas replicate PCR runs were used to estimate *p*, each individual bird was sampled on a single occasion, and therefore our dataset did not contain pseudo-replicates for analysis of Ψ. Occupancy modeling was conducted using Program MARK [64].

We developed an a priori suite of models to assess temporal variation in hematozoa prevalence and detection on the breeding grounds. Because species were sampled unequally during each of the three capture periods (Table 1), we modeled prevalence by monthly sampling period (May, June, August) and by species, sex, and age class within each month. To limit the total number of models considered, we conducted model selection for each parasite genus using a 3-staged approach; the top-supported model from each stage was carried on to the succeeding stage where it was used as base structure for consideration of additional variables. Relative support among models in each stage was assessed using Akaike’s Information Criterion corrected for sample size (AIC_c_). Models were not considered further if their AIC_c_ values were greater than (i) a nested model with fewer parameters, or (ii) a more parameterized model with similar structure, but one additional parameter [65, 66]. In Stage 1, we assessed general ecological variation in detection probability and hematozoa prevalence by considering variation relative to duck species, age, sex, and month; in Stage 2, we considered models assessing relationships between genus-specific prevalence and co-infection with other parasite genera and IAV; and finally in Stage 3, we examined the effects of size-adjusted body condition on parasite infection and co-infection.

Peaks in abundance of parasitemia (i.e. parasite load) in avian hosts occur following initial infection, and to a lesser extent upon recrudescence associated with the spring breeding period in years subsequent to initial infection [1]. During the latent period in between these peaks, parasitemia in blood are reduced to low levels, perhaps below the threshold of detection via microscopy or molecular methods [7, 67, 68]. As such, we suspected that peaks in parasitemia for individuals may be correlated with seasonal peaks in population-level prevalence as a function of a positive relationship between molecular detection of hematozoa parasites and host parasite load [69]. Therefore, although our primary interest was in obtaining unbiased estimates of Ψ, in practice, *p* and Ψ may have been correlated such that variation in both variables were likely to explain variation in true prevalence [70]. To account for potential variation in detection probability, we first considered a range of models to identify the top approximating structure for *p*. Specifically, we constrained Ψ to a highly parameterized structure while considering each of the models from Stage 1 on *p* (Table 2). We then fixed *p* to the top supported structure for modeling of Ψ in each subsequent stage. For cases in which all PCR runs for a given class of birds were positive for parasite mtDNA (i.e. all encounter histories 1,1), *p* was fixed to 1.0. Due to the low frequency of *Plasmodium* parasites in samples collected during May and June, we limited sources of variation considered on *p* for *Plasmodium* to month and species during these 2 sampling periods (Additional file 1: Table S9). Reported estimates of *p* correspond to a single PCR run, thus detection probability for duplicate runs is calculated as: 1 – (1 – *p*)^2^.

**Table 2:** Suite of models considered in Stage 1 to estimate probability of hematozoa infection (Ψ) and probability of detecting hematozoa mtDNA (*p*)

Model	K	Model description
(.)	1	pr (infection) does not vary
month	3	pr (infection) varies by month (May, June, August)
May models (17 May – 27 May)		
sex	2	pr (infection) varies during May by sex
species	4	pr (infection) varies during May by species (MALL, NOPI, AMWI, LESC)
species + sex	5	pr (infection) varies during May by species and by sex to the same degree for all species
June models (10 July – 19 June)		
sex	2	pr (infection) varies during June by sex
species	3	pr (infection) varies during June by species (MALL, NOPI, AMWI)
species + sex	4	pr (infection) varies during June by species and by sex to the same degree for all species
August models (28 July – 20 August)^a^		
age	2	pr (infection) varies during August by age (adult, juvenile)
sex	2	pr (infection) varies during August by sex
age + sex	3	pr (infection) varies during August by age and by sex to the same degree for both age classes
age * sex	4	pr (infection) varies during August by each age and sex class
(ad * sex) + (juv)	3	pr (infection) varies during August by age and by sex for adult birds
species	3	pr (infection) varies during August by species (MALL, NOPI, AGWT)
species * age	6	pr (infection) varies during August by species and age
species + age	4	pr (infection) varies during August by species and by age to the same degree for all species
species * sex	6	pr (infection) varies during August by species and sex
species + sex	4	pr (infection) varies during August by species and by sex to the same degree for all species
species * age + sex	7	pr (infection) varies during August by species and age, and by sex to the same degree for all species
species * sex + age	7	pr (infection) varies during August by species and sex, and by age to the same degree for all species
species * age * sex	12	pr (infection) varies during August by species, age, and sex
(ad * species * sex) + (juv * species)	9	pr (infection) varies during August by species and by sex for adult birds
August temporal trend models		
+ day	1	pr (infection) during August varies in a linear trend with a single slope for all sources of variation
+ day * age^b^	2	pr (infection) during August varies in a linear trend with a unique slope for each age class
* day	2+	pr (infection) during August varies in a linear trend with a unique slope for each source of variation

This suite of models was considered independently for each of three hematozoa genera (*Leucocytozoon*, *Haemoproteus*, *Plasmodium*). K = number of model parameters applicable only to modeling of Ψ or *p* in a given month and is not representative of the total number of model parameters. Age of birds was either after-hatch-year (adult) or hatch-year (juvenile). Species *abbreviations*: *AGWT* American green-winged teal, *AMWI* American wigeon, *LESC* lesser scaup, *MALL* mallard, *NOPI* northern pintail

^a^August models were considered alone, with the additive temporal trend (+ day), with the temporal trend varying by age (+ day * age), and with the multiplicative temporal trend (* day)

^b^Model structure was applied only to August models containing variation in age

To assess temporal variation in prevalence, we modeled Ψ separately for each month (May, June, August) and assessed month-specific variation relative to species, sex, and age. We expected seasonal prevalence in adult birds to be highest towards the beginning of the breeding season as a result of recrudescence and new infections, and then subsequently decline with seasonal progression as infections were cleared by host immune response, but we suspected that prevalence in juvenile birds would exhibit a different pattern, peaking later in the season than adults due to delayed exposure to vectors [71, 72]. Our sample during the May and June intervals was limited to adult birds, and was weighted towards males, so we considered models in which Ψ was constant or varied relative to species, sex, or by species with an additive effect of sex. During August our sample contained both adult and juvenile birds and more equal representation of sexes, so we considered variation relative to species, age, sex, and the multiplicative and additive effects of each variable. Whereas hematozoa prevalence may vary between sexes of adult birds due to differences in behavior influencing exposure to vectors during the breeding season, or to sex-specific factors affecting immunocompetence [73], we predicted that prevalence for juveniles would not differ by sex given that young of both sexes remain in close proximity throughout the brood-rearing period and have similar behavior during autumn staging. To test this hypothesis, we considered an additional form of variation by age and sex in which prevalence varied by sex for adults, but was constrained to be equal between sexes for juvenile birds ((ad * sex) + (juv)). Finally, we assessed support for temporal variation within the August sampling interval by considering a linear trend in prevalence by sampling day (28 July = 1, 20 Aug = 24) to assess directionality in temporal trends of prevalence. We considered a suite of 14 models assessing variation in prevalence relative to species, age, and sex, and to each of these we considered an additive temporal trend, a temporal trend varying by age, and a temporal trend varying relative to each term in a given model. Our model set in Stage 1 contained 60 models on each of *p* and Ψ (Table 2).

In Stage 2 of model selection, we assessed the relationship between probability of infection with a given parasite genus and other parasite genera, active IAV infection, and previous IAV infection (serostatus) to ascertain potential interactions among infectious agents. Specifically, co-infection with each hematozoa genus, IAV, and serostatus were coded as binomial individual covariates. We considered models where each co-infection variable was added to the top-supported model from Stage 1 as a constant effect, and varying relative to month, species, age, and sex. The nested-PCR protocol that we used employed a single primer set for *Haemoproteus* and *Plasmodium* parasites, making distinguishing between the two genera in a single host difficult [74]. Thus, we were less likely to detect co-infections with *Haemoproteus* and *Plasmodium*, and therefore did not consider these relationships in our models. When modeling *Leucocytozoon* prevalence, we considered an additional suite of 20 models in Stage 2, whereas for *Haemoproteus* and *Plasmodium*, we considered 15 additional models (Table 3). We hypothesized that hematozoa and IAV prevalence may be positively correlated if immunosuppression resulting from one infection increased susceptibility of ducks to another infection. Additionally, we assessed the hypothesis that previous exposure to IAV, measured based on the presence of IAV antibodies, may reduce susceptibility to hematozoa infection as was recently demonstrated with West Nile Virus and *Plasmodium* in songbirds [36].

**Table 3:** Suite of models considered in Stage 2 and Stage 3 to estimate probability of hematozoa infection (Ψ)

Model	K	Model description
Stage 2 – Co-infection models^a^		
Stage 1	0	pr (infection) varies by top supported model structure from Stage 1
Haem	1	pr (infection) varies by Stage 1 and by co-infection with *Haemoproteus* parasites
Leuc	1	pr (infection) varies by Stage 1 and by co-infection with *Leucocytozoon* parasites
Plas	1	pr (infection) varies by Stage 1 and by co-infection with *Plasmodium* parasites
IAV	1	pr (infection) varies by Stage 1 and by co-infection with Influenza A Virus
Serostatus	1	pr (infection) varies by Stage 1 and by Influenza A Virus serostatus
Co-infection variation		
* month	3	pr (infection) varies by Stage 1 and by co-infection differently for each month
* species	5	pr (infection) varies by Stage 1 and by co-infection differently for each duck species
* age	2	pr (infection) varies by Stage 1 and by co-infection differently for each age class
* sex	2	pr (infection) varies by Stage 1 and by co-infection differently for each sex class
Stage 3 – Body condition models		
Stage 2	0	pr (infection) varies by top supported model structure from Stage 2
BCI	1	pr (infection) varies by Stage 2 and by body condition
BCI * month	3	pr (infection) varies by Stage 2 and by body condition effect that is different for each month
BCI * species	5	pr (infection) varies by Stage 2 and by body condition effect that is different for each species
BCI * age	2	pr (infection) varies by Stage 2 and by body condition effect that is different for each age class
BCI * sex	2	pr (infection) varies by Stage 2 and by body condition effect that is different for each sex class

Models were considered independently for each of three hematozoa genera (*Leucocytozoon*, *Haemoproteus*, *Plasmodium*) and were applied only to estimation of Ψ. K = number of model parameters in addition to those supported in the previous stage of model selection and BCI is the body condition index

^a^Each co-infecting agent was considered alone, and varying multiplicatively with co-infection variation structures

Finally, in Stage 3, we assessed support for variation in hematozoa prevalence relative to body condition to make inference on possible sub-lethal effects of infection. Our measure of body condition index (BCI) was size-adjusted body mass. We controlled for known sources of variation in body mass of ducks (i.e. species, age, sex, and seasonal timing) by calculating the BCI variable separately for each species, age, and sex class by month; this approach allowed for direct assessment of relationships between body condition and hematozoa prevalence in a single parameter. For each class, we performed a principal components analysis on the correlation matrix for lengths of head and tarsus, regressed body weight (g) on PC1 scores, and then divided residuals from the regression by class-specific mean weight. Hence, inference regarding the relationship between BCI and Ψ apply to the class-specific relationships. Analyses to construct the BCI variable were conducted using SAS (SAS Institute 1996). Deleterious effects from hematozoa infection are likely strongest during temporal periods associated with primary parasitemia, and may be greater in young and newly infected hosts [1]. Therefore, we considered models in which BCI was either additive (i.e. a single parameter), or varied relative to month, species, age, and sex (Table 3). To assess potential patterns in body condition associated with co-infections, we also considered variation in BCI relative to any supported co-infection variables from Stage 2 (Table 3).

We report coefficient estimates using 85 % confidence intervals because they are more compatible with an AIC-based modeling approach than traditional 95 % confidence intervals [65]. Estimates of prevalence and detection probability were back-transformed from the logit link and associated variances were calculated using the delta method. We report estimates ± SE unless otherwise stated.

## Results

### Detection of hematozoa, IAV, IAV antibodies, and co-infections

We detected infection by hematozoa parasites in 775 of the 1347 ducks sampled (57.5 %); apparent prevalence was 43.7 % for *Leucocytozoon*, 20.9 % for *Haemoproteus*, 13.3 % for *Plasmodium*, and 9.7 % for IAV. Antibodies to IAV were detected in 21.7 % of samples. Co-infection of *Leucocytozoon* and *Haemoproteus* parasites occurred in 196 birds, whereas 78 birds were co-infected with *Leucocytozoon* and *Plasmodium* parasites. We detected only two co-infections containing *Haemoproteus* and *Plasmodium* parasites, which is likely, at least in part, a function of molecular methods employed (see Methods). Seventy-two birds were identified as being infected with hematozoa and actively shedding IAV, and 196 birds that were infected with blood parasites also tested positive for IAV antibodies.

Sources of variation in detection probability differed among parasite genera (Table 4). Detection of *Plasmodium* was constant across all months (0.86 ± 0.02), but detection varied by month, species, and age for *Haemoproteus* and by month, age, and sex for *Leucocytozoon* (Table 5). Detection of *Haemoproteus* ranged from 0.85 (±0.03) to 0.98 (±0.01) for adult birds, but was lower for juveniles among mallards (0.41 ± 0.13), northern pintails (0.69 ± 0.09), and green-winged teal (0.81 ± 0.11). Detection of *Leucocytozoon* in May was 0.90 (±0.02) and in June was 0.97 (±0.02). During August, detection of *Leucocytozoon* increased linearly through time for adult female (range: 0.80 ± 0.05–0.90 ± 0.05), adult male (range: 0.67 ± 0.07–0.99 ± 0.01), and juvenile (range: 0.74 ± 0.07–0.93 ± 0.02) ducks.

**Table 4:** Top AIC_c_ approximating model explaining variation in detection probability (*p*) and probability of infection (Ψ) for three hematozoa genera following three stages of model selection

		Top approximating model	
Parasite genus	Model stage	*p*	Ψ
Leuc	Stage 1	*p*_May_(.) + *p*_Jun_(.) + *p*_Aug_((ad * sex) + juv) * day)	Ψ_May_(spp + sex) + Ψ_Jun_(spp) + Ψ_Aug_(spp * age) + (age * day))
	Stage 2	--	Stage 1 + Haem
	Stage 3	--	Stage 2 + (BCI * spp)
Haem	Stage 1	*p*_May_(.) + *p*_Jun_(.) + *p*_Aug_(spp + age)	Ψ_May_(spp + sex) + Ψ_Jun_(spp) + Ψ_Aug_((ad * spp * sex) + (juv * spp))
	Stage 2	--	Stage 1 + Leuc
	Stage 3	--	Stage 2 + (BCI * age)
Plas	Stage 1	*p*(.)	Ψ_May_(sex) + Ψ_Jun_(spp + sex) + Ψ_Aug_((spp + age) * day)
	Stage 2	--	Stage 1
	Stage 3	--	Stage 2

Model structure explaining variation in *p* was fixed following Stage 1 for additional investigation of variation on Ψ in Stages 2 and 3. *Abbreviations*: Leuc *Leucocytozoon*, Haem *Haemoproteus*, Plas *Plasmodium*, *spp* duck species, *ad* age class adult, *juv* age class juvenile

**Table 5:** Estimates of monthly detection probability $\left(\widehat{p}\right)$ for *Leucocytozoon*, *Haemoproteus*, and *Plasmodium* parasites for a single PCR run

		May		June		August	
Parasite	Class	$\widehat{p}$	SE	$\widehat{p}$	SE	$\widehat{p}$	SE
*Leucocytozoon*	All	0.90	0.02	0.97	0.02		
	ad - M					0.67, 0.93, 0.99	0.07, 0.02, 0.01
	ad - F					0.80, 0.85, 0.90	0.05, 0.03, 0.05
	juv					0.74, 0.86, 0.93	0.07, 0.02, 0.02
*Haemoproteus*	All	0.85	0.03	0.85	0.04		
	AGWT - ad					0.98	0.01
	MALL - ad					0.91	0.03
	NOPI - ad					0.97	0.02
	AGWT - juv					0.81	0.11
	MALL - juv					0.41	0.13
	NOPI - juv					0.69	0.09
*Plasmodium*	All	0.86	0.02	(applies to all months)			

Ducks were sampled in three distinct capture intervals: May (17 May – 27 May), June (10 June – 19 June), and August (28 July – 20 August). Estimates of *p* for *Leucocytozoon* increased linearly during the August sampling period; numbers represent the lowest, median, highest estimates and associated SE. *Abbreviations*: *ad* age class adult, *juv* age class juvenile, *MALL* mallard, *NOPI* northern pintail, *AGWT* American green-winged teal

### Hematozoa prevalence

**Fig. 2:**
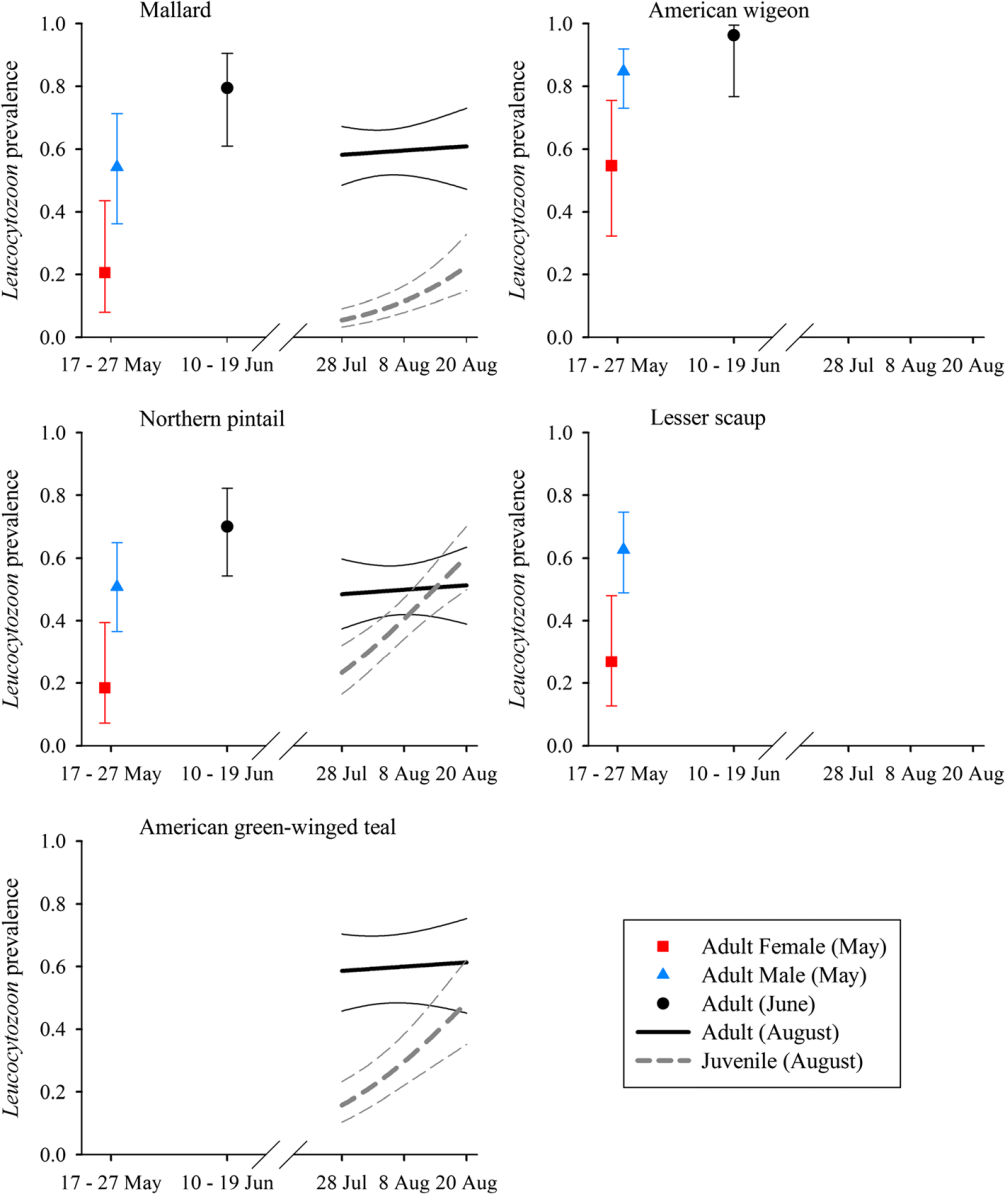
Estimated prevalence (±95 % CI) of *Leucocytozoon* parasites infecting five duck species sampled during May – August, 2010, interior Alaska

*Leucocytozoon* prevalence varied by host species and sex during May, by species during June, and by species, age, and time during August (Table 4). Estimates of *Leucocytozoon* prevalence were as low as 0.05 (±0.01) for juvenile mallards during the beginning of August and as high as 0.96 (±0.04) for adult American wigeon during June (Fig. 2). *Leucocytozoon* prevalence increased from May to June and was lower during August than June for adult birds. Estimated prevalence during the August sampling period increased strongly for juvenile mallards (28 Jul: 0.05 ± 0.01, 20 Aug: 0.23 ± 0.05), green-winged teal (28 Jul: 0.16 ± 0.03, 20 Aug: 0.48 ± 0.07), and northern pintails (28 Jul: 0.23 ± 0.04, 20 Aug: 0.60 ± 0.05), and increased slightly for adults of these three species (Fig. 2). Estimated prevalence of *Leucocytozoon* parasites was consistently higher for mallards than northern pintails in all three months, and *Leucocytozoon* prevalence for American wigeon exceeded those of other species during May and June, the two months for which data were obtained for this species (Fig. 2). Prevalence of *Leucocytozoon* for adult mallards, northern pintails, and green-winged teal during August was higher than for juvenile ducks of respective species, although these differences were largest early in the sampling period (Fig. 2).

**Fig. 3:**
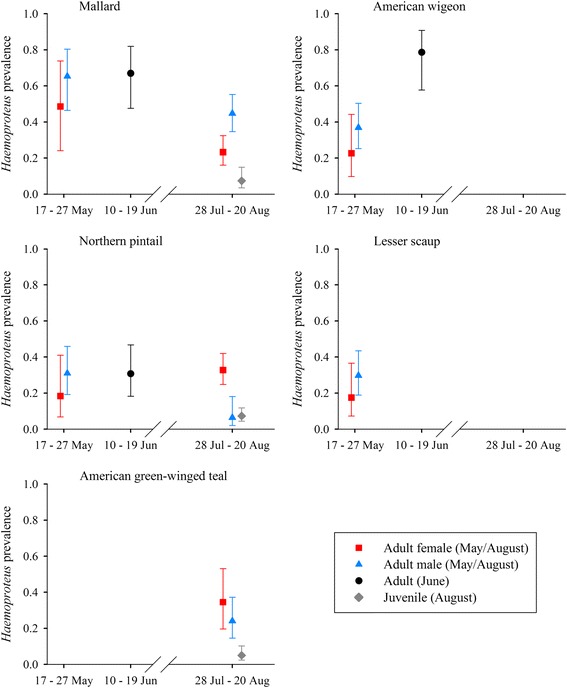
Estimated prevalence (±95 % CI) of *Haemoproteus* parasites infecting five duck species sampled during May – August, 2010, interior Alaska

Prevalence of *Haemoproteus* parasites varied by host species and sex during May, by species during June, and by species, age, and adult sex during August (Table 4). Estimates of *Haemoproteus* prevalence were as low as 0.05 (±0.02) for juvenile green-winged teal and as high as 0.79 (±0.08) for adult American wigeon during June. Estimates of *Haemoproteus* prevalence were relatively constant from May to June for mallards (May females: 0.49 ± 0.14, May males: 0.65 ± 0.09, June: 0.67 ± .09) and northern pintails (May females: 0.18 ± 0.09, May males: 0.30 ± 0.07, June: 0.31 ± 0.07); however, for American wigeon, prevalence during June (0.79 ± 0.08) was considerably higher than May estimates for both females (0.23 ± 0.09) and males: (0.37 ± 0.07). We did not find strong support for temporal trends in prevalence of *Haemoproteus* during the August sampling period (ΔAIC_c_ = 0.95, Additional file 1: Table S6), and species and sex-specific estimates were generally lower during August than in previous months (Fig. 3). *Haemoproteus* prevalence for adult mallards, northern pintails, and green-winged teal exceeded that of juvenile birds during August, similar to results for *Leucocytozoon* (Fig. 2).

**Fig. 4:**
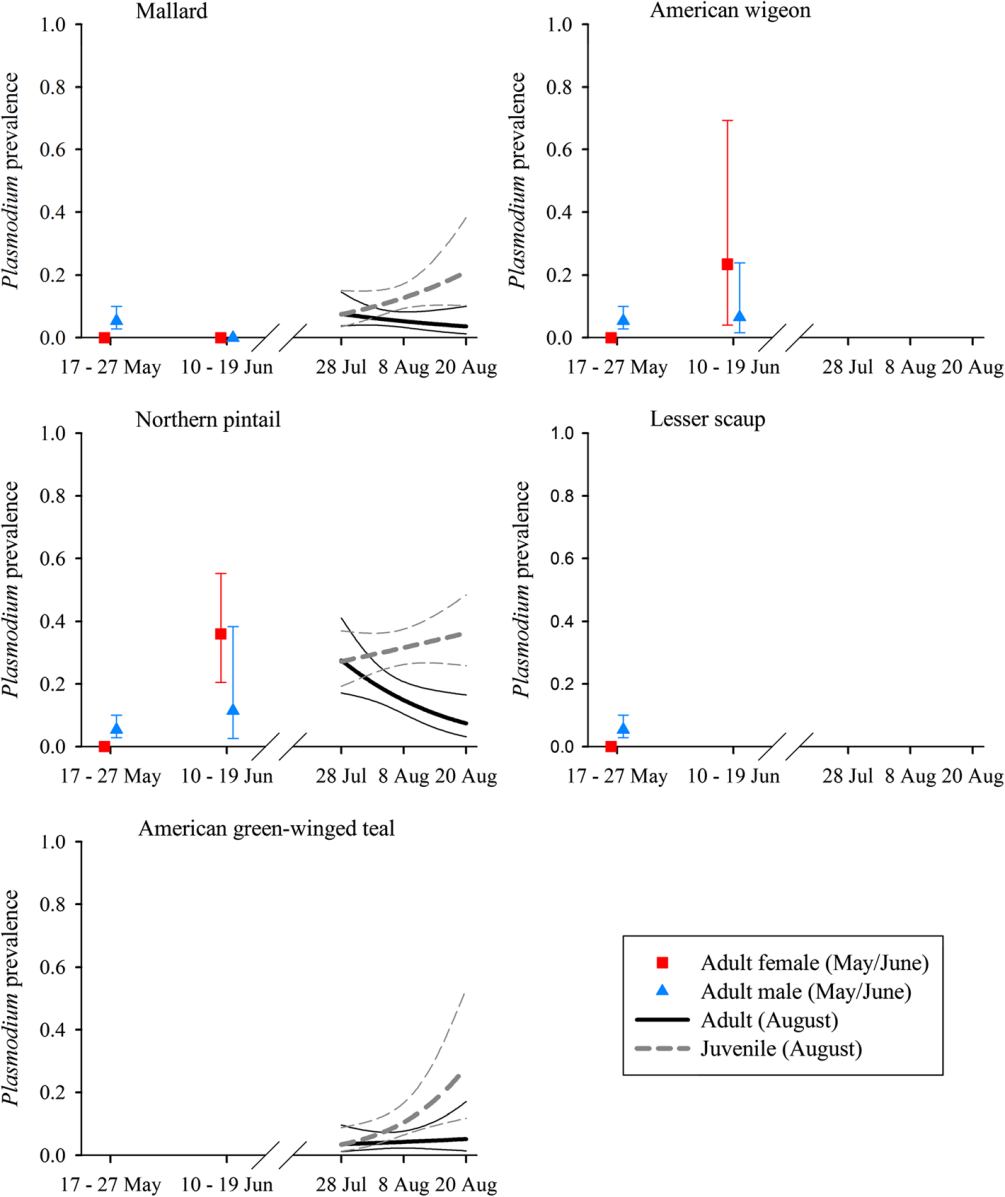
Estimated prevalence (±95 % CI) of *Plasmodium* parasites infecting five duck species sampled during May – August, 2010, interior Alaska

**Fig. 5:**
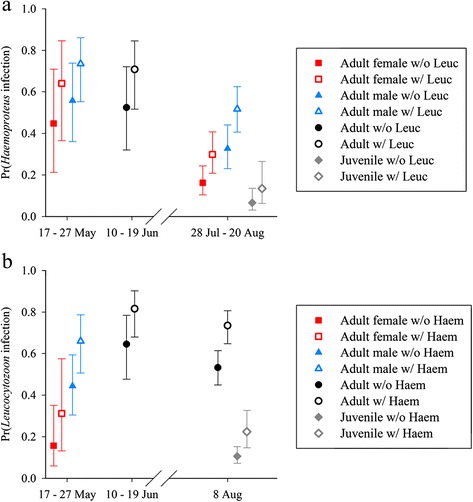
Co-infection relationships between infection status with *Haemoproteus* and *Leucocytozoon* parasites in northern pintails. Panel **a** depicts estimated probability of infection with *Haemoproteus* parasites (±95 % CI) relative to *Leucocytozoon* (Leuc) infection status; panel **b** depicts estimated probability of infection with *Leucocytozoon* parasites (±95 % CI) relative to *Haemoproteus* (Haem) infection status. Estimates were produced from the top approximating genus-specific models in Stage 2 of model selection

Prevalence of *Plasmodium* parasites varied by sex during May, by species and sex during June, and by species, age, and day during August (Table 4). *Plasmodium* prevalence ranged from a lack of detection in any species of female duck during the May sampling period, or in mallards during June (i.e. 0.00), to as high as 0.36 (±0.06) for juvenile northern pintails at the end of the August sampling period (Fig. 4). We did not find strong support for variation in *Plasmodium* prevalence among species during May (ΔAIC_c_ = 2.67, Additional file 1: Table S10), but prevalence for northern pintails was higher during June (female: 0.36 ± 0.09; male: 0.11 ± 0.08) than for American wigeon (female: 0.23 ± 0.18, male: 0.07 ± 0.05) and mallards (0.00), and mean prevalence during August was higher for both adult (0.17 ± 0.03) and juvenile (0.31 ± 0.03) northern pintails than for mallards (adult: 0.06 ± 0.01; juvenile: 0.13 ± 0.02) and green-winged teal (adult: 0.04 ± 0.01; juvenile: 0.09 ± 0.02). During the August sampling period, *Plasmodium* prevalence increased for juvenile mallards, northern pintails, and green-winged teal, but decreased or remained stable during August for adult ducks of these three species (Fig. 2). In contrast to results for *Leucocytozoon* and *Haemoproteus*, prevalence of *Plasmodium* was higher for juvenile than adult ducks during August (Fig. 2).

### Co-infections

We found strong support for a positive relationship between co-infections with *Leucocytozoon* and *Haemoproteus* parasites (Additional file 1: Tables S3 and S7). Estimated prevalence of *Leucocytozoon* was 1.04–2.12 times higher given co-infection with *Haemoproteus* (${\widehat{\beta}}_{Haem}=0.89$, 85 % CI = 0.64, 1.14, Additional file 2: Table S13), whereas estimated prevalence of *Haemoproteus* was 1.25–2.10 times higher given co-infection with *Leucocytozoon* (${\widehat{\beta}}_{Leuc}=0.79$, 85 % CI = 0.55, 1.03, Additional file 2: Table S14), depending upon month, duck species, age, and sex (Fig. 5). We did not find support for any relationships between infection with *Leucocytozoon* and *Plasmodium*, and there was no support for relationships between infection with any parasite genus and IAV (Table 4, Additional file 1: Tables S3, S7, and S11).

### Hematozoa infection and body condition

We found support for variation in prevalence relative to BCI for *Leucocytozoon* and *Haemoproteus*, but not for *Plasmodium* (Table 4). The BCI effect for *Haemoproteus* was negative for juvenile birds (${\widehat{\beta}}_{BCI(juv)}=-5.29$, 85 % CI = -8.72, -1.87) and weakly negative for adults (${\widehat{\beta}}_{BCI(ad)}=-1.08$, 85 % CI = -2.71, 0.56). When comparing the probability of *Haemoproteus* infection across the spectrum of BCI values for juvenile mallards, northern pintails, and green-winged teal, estimated probabilities of infection for ducks in the lowest 10^th^ percentile of body condition were 2.36–3.24 times higher than for ducks in the highest 90^th^ percentile (Additional file 2: Table S15). For *Leucocytozoon*, the BCI effect varied by species and was negative for northern pintails (${\widehat{\beta}}_{BCI(NOPI)}=-3.25$, 85 % CI = -4.95, -1.54), positive for American wigeon (${\widehat{\beta}}_{BCI(AMWI)}=15.46$, 85 % CI = 6.10, 24.83), and equivocal for lesser scaup (${\widehat{\beta}}_{BCI(LESC)}=-0.38$, 85 % CI = -7.37, 6.60), green-winged teal (${\widehat{\beta}}_{BCI(AGWT)}=-0.50$, 85 % CI = -3.22, 2.21), and mallards (${\widehat{\beta}}_{BCI(MALL)}=1.23$, 85 % CI = -0.96, 3.42). The probabilities of *Leucocytozoon* infection for northern pintails in the lowest 10^th^ percentile of body condition were 1.26–1.62 times higher than for those in the highest 90^th^ percentile, whereas American wigeon in the highest 90^th^ percentile of body condition were 1.04–3.48 times more likely to be infected than those in the lowest 10^th^ percentile (Additional file 2: Table S16). We did not find support for variation in prevalence relative to interactions with co-infection status and BCI (Additional file 1: Tables S4 and S8).

## Discussion

In this study we screened a large number of waterfowl samples collected in the northern sub-Arctic and found relatively high prevalence of three genera of hematozoa parasites in adults and juveniles of five species of ducks, which provides evidence for local transmission. Molecular detection of hematozoa varied by parasite genus and a suite of host traits, but was relatively high, especially given two replicate PCR runs of each sample, indicating that we were successfully able to detect the majority of infections and reduce potential bias in estimates of prevalence. Parasite genus, seasonal timing, and duck species and age were important sources of variation in hematozoa prevalence, providing evidence for complex epidemiological patterns of infection. Co-infections with multiple genera of blood parasites were common, as was evidence for infection with hematozoa and current or past infection with IAV, although only the positive relationship between *Leucocytozoon* and *Haemoproteus* infection was correlated with the probability of infection of a given parasite genus. Finally, an assessment of relationships between size-adjusted body mass and infection status revealed a negative relationship between body condition and infection with *Haemoproteus*, but this relationship varied by duck species for *Leucocytozoon* infection, revealing no clear patterns.

The heterogeneity in detection probability observed in this study highlights the importance of tailoring protocols to allow for estimation of false negatives (i.e. through multiple PCR runs per blood sample) to minimize potential sources of bias in estimates of prevalence. Detection probability of hematozoa mtDNA using molecular methods has previously been shown to vary relative to parasite genus, geographic location, and host age [13, 14], which may be the result of variation in parasite load among host groups [69]. We found support for variation in detection probability by season, age, sex, and species, although for adult ducks, estimates of detection of all genera of parasites from a single PCR run were ≥ 0.85, and therefore the probability of detecting infection with two replicate runs (1 – (1 – *p*)^2^) was at least 0.98. Variation in detection for juvenile ducks was more pronounced, especially for *Haemoproteus* parasites, where estimates of detection were considerably lower for juveniles than for adults in their respective species $\left({\widehat{\rho}}_{\mathrm{juv}\;\mathrm{mallards}}=0.41;\;{\widehat{\rho}}_{\mathrm{juv}\;\mathrm{northern}\;\mathrm{pintails}}=0.69;\;{\widehat{\rho}}_{\mathrm{juv}\;\mathrm{green}\hbox{-} \mathrm{winged}\;\mathrm{teal}}=0.81\right)$. Hence, while our results indicate that duplicate PCR runs sufficiently decreased the likelihood of false negatives in our data for adult birds, the probability of detecting *Haemoproteus* infections in juveniles using duplicate runs was still as low as 0.65, 0.90, and 0.96 for mallards, northern pintails, and green-winged teal, respectively. The use of triplicate runs would allow for more sophisticated models that recognize individual heterogeneity in detection probability, possibly related to intensity of infection, and may be warranted in studies where high levels of precision on estimates of detection probability are critical towards achieving project objectives, or when it is necessary to substantially reduce sources of bias in estimates of prevalence.

For adult birds of all species investigated in this study, *Leucocytozoon* prevalence was the highest, followed by *Haemoproteus* and *Plasmodium*, however, for juvenile ducks, prevalence of *Leucocytozoon* and *Plasmodium* exceeded that of *Haemoproteus*. Similar patterns have been reported in previous investigations of North American waterfowl [19, 75] and in recent high-latitude investigations of waterfowl [13, 14] and songbirds [21]. Our mean species-specific estimates of *Plasmodium* prevalence in juvenile ducks (range: 0.08 – 0.31), which are indicative of local transmission, are considerably higher than estimates of juvenile (0.01) and resident adult (0.05) songbirds at a nearby location of the same latitude [17]. These differences may be due to variation in susceptibility or exposure of waterfowl versus passerines, may reflect relatively high densities of mosquito vectors in the Minto Flats wetlands where we sampled, or could be a function of inter-annual variation in hematozoa prevalence [55, 72].

Our results indicate that, for adult birds, prevalence of all three hematozoa genera is highest during May and June, with peaks in prevalence occurring in association with pre-nesting and breeding, and diminishing during the autumn staging period in August. In contrast, our results indicate increasing trends in prevalence for *Leucocytozoon* and *Plasmodium* parasites during August for juvenile ducks. Contrasting patterns in seasonal prevalence by age class is likely due to differential timing of exposure to vectors and recrudescence from previous infections in adults [72]. That is, adult birds may experience re-lapse from previous infections prior to or shortly after arrival on the breeding grounds, and acquire new infections through exposure to vectors during the breeding period. However, juvenile birds in our study hatched during June and July and were therefore exposed to vectors of hematozoa later in the season. Duration of hematozoa infection in wild waterfowl is not well understood, but we suspect that many adult ducks may have been clearing infections at the time that juvenile ducks were first susceptible to infection. Higher prevalence in adults may be expected given that prevalence of juveniles represents only infections obtained in the given year, whereas prevalence in adult ducks represents new infections plus those retained from previous seasons. For the three duck species sampled when juveniles were available for capture (i.e. mallard, northern pintail, green-winged teal), *Haemoproteus* and *Leucocytozoon* prevalence was higher for adults, while prevalence of *Plasmodium* was up to several times higher for juvenile birds of these same three species. This suggests that juvenile ducks may be more susceptible to *Plasmodium* infections than adults, *Plasmodium* infections take longer for juveniles to clear, or encounter rates with mosquito vectors vary by age class. It is plausible that downy feathers of ducklings provide less defense against biting vectors than the fully developed feathers of adult waterfowl [13], although given such high rates of *Plasmodium* infection in juvenile birds, it is surprising that recrudescence of *Plasmodium* infections in adults was not apparent through higher estimates of prevalence. This may be a result of the peak of recrudescence occurring prior to our sampling in mid-May, or could, at least in part, be an artifact of the molecular approach used to detect infections. That is, we used the same primers to amplify parasites from both *Haemoproteus* and *Plasmodium* genera, hence, it is plausible that in some cases where birds were co-infected with parasites of both genera, only *Haemoproteus* infection was detected, thus resulting in negatively biased estimates of *Plasmodium* prevalence. Future investigations could provide further inference by incorporating samples collected earlier in the breeding season, developing molecular tests that use different primers for *Haemoproteus* versus *Plasmodium* parasites, and by incorporating microscopy that may be useful for identifying co-infections based upon morphological examination of thin blood smears.

We did not find evidence that hematozoa prevalence varied relative to sex for juvenile ducks; however, for adults, variation in prevalence by sex was dependent upon seasonal timing and parasite genus. Differences in prevalence by sex in adult birds may be due to sex-specific differences in host immunocompetency or to differences in behavior leading to differential exposure to vectors [73]. Male and female ducks exhibit considerably different behaviors during the breeding period; upon clutch completion, male ducks depart the nest site to undergo remigial molt whereas females incubate eggs and rear the young [76]. Whereas our estimates of hematozoa prevalence were higher for males than females for all species of ducks and parasite genera during May, differences in *Haemoproteus* prevalence among species by sex did not exhibit consistent patterns in June or August. Thus, the mechanisms driving sex-specific variation in parasite prevalence are unclear and represent an important topic for further investigation.

Variation in hematozoa prevalence among co-occurring avian host species has been frequently reported, and a number of hypotheses have been put forth to explain such findings. These hypotheses include differential exposure to vectors resulting from timing of nest initiation [77] or habitat selection [28], differential vector preference relative to host body size in which larger birds attract more vectors [29], or to plumage characteristics in which vectors are attracted to brighter colors [78]. In comparative studies, Ricklefs [30] and Tella et al. [79] demonstrated that prevalence was inversely related to the relative length of the incubation period, presumably because slower growth periods associated with prolonged incubation leads to more robust immunocompetency. We did not directly assess variation in prevalence relative to timing of nest initiation or species-specific incubation periods, and our sampling design did not allow for direct assessment of body size effects due to confounding of new infections and recrudescence among adults, and the correlation between juvenile body size and time, but there does not appear to be evidence in our data to support these hypotheses. Green-winged teal were the smallest species sampled in our study (mean mass = 314 g) and had the shortest incubation period (~22 days) [80], yet hematozoa prevalence of green-winged teal was generally similar to, or on occasion higher than, that of mallards, the species with the largest body size (mean mass = 1100 g) and the longest incubation period (~28 days) [80]. Furthermore, species-specific estimates of *Leucocytozoon* prevalence during May and June, and of *Haemoproteus* prevalence during June, were highest for American wigeon, a species of intermediate size (mean mass = 708 g) and incubation period (~25 days) [80]. Variation in behavior or habitat selection among species that influences exposure to vectors may account for species-specific differences in prevalence, but throughout the duration of sampling, we frequently captured multiple species simultaneously, indicating substantial overlap in habitat use. Thus, although we cannot rule out mechanisms associated with host or vector behavior on our study area, we suspect that variation in prevalence among species in our study was likely due either to temporal or geographical variation in migratory patterns leading to differential exposure to vectors off of the breeding grounds, or to species-specific disparities in host immunocompetence [6, 30, 31, 81].

Existing parasitic infections in a host may increase the likelihood of a secondary infection resulting from immunosuppression or down-regulation of the host immune system, or, existing infections may limit secondary infections due to competitive exclusion or elicitation of cross-protective immune response [9, 82]. In our study, co-infections with *Leucocytozoon* and *Haemoproteus* parasites occurred together three times more frequently than expected given the relative prevalence of each genus, and this positive relationship between infection with *Leucocytozoon* and *Haemoproteus* is consistent with results of Arriero and Moller [32] and Van Rooyen et al. [34], who speculated that *Leucocytozoon* parasites primarily infect hosts with existing infections. Whereas prevalence of both *Leucocytozoon* and *Haemoproteus* varied considerably by month and among species and ages in our study, we did not find support for variation in the co-infection relationships relative to any of these factors, indicating a high degree of constancy in the mechanisms associated with co-occurrence of these parasite genera. This relationship may also be due to host traits in that an individual behavior leading to exposure to vectors of one genus may, for the same reason, lead to exposure to vectors for the other genus. Whereas black flies (Diptera: Simuliidae) and biting midges (Diptera: Ceratopogonidae) are generally considered to be the genus-specific vectors of *Leucocytozoon* and *Haemoproteus*, respectively, knowledge of vectors in Alaska is largely lacking. Therefore, it may be plausible that in our study area, both *Leucocytozoon* and *Haemoproteus* parasites are transmitted by the same vector. Alternatively, genus-specific vectors may utilize similar habitats such that ducks exposed to one are more likely to be exposed to the other. In a study of hematozoa co-infection relationships among songbirds in the Boreal forest region of Alaska, Oakgrove et al. [21] observed a negative relationship between *Leucocytozoon* and *Haemoproteus* infections and suggested that this result may be due to within-host competition among parasite lineages. This discrepancy in findings is unexpected given the close proximity of our study site with theirs, but may be due to species differences in parasites infecting waterfowl versus those infecting songbirds [1, 57].

We found no relationships between active or previous IAV infection and the probability of hematozoa infection, suggesting that the mechanisms influencing infection of IAV in waterfowl on the breeding grounds are independent of those influencing susceptibility to hematozoa. Aquatic birds, and waterfowl in particular, are the primary reservoir for IAV, and it is generally accepted that the viruses circulating in wild duck populations cause minimal deleterious effects on waterfowl hosts [83]. Thus, the avirulent nature of endemic IAVs may not illicit a strong enough effect on the host immune system to influence susceptibility to hematozoa. Likewise, host immune response to infection by endemic hematozoa lineages may be insufficient to influence susceptibility to IAV, or the contrasting epidemiological characteristics of IAV and hematozoa (e.g. differential route of infection, lack of within-host competition) may prohibit cross-protective immunological properties. It is plausible that other infectious agents (e.g. viruses, bacteria, intestinal parasites, ectoparasites) influence the susceptibility and transmission of hematozoa in waterfowl and future research may be warranted.

Similar to an investigation of nesting wild ducks in Saskatchewan, Canada [40], we observed a negative relationship between body condition and infection status with *Haemoproteus* parasites, although the effect in our study was strongest for juvenile ducks. This may be indicative of a positive relationship between body condition and host immunocompetence, or conversely, costs of *Haemoproteus* infection may result in reductions to host body condition. We suspect that the relationship between body condition and *Haemoproteus* infection was strongest in juvenile ducks due to the relative parasite load and associated immune response in primary versus secondary parasitemia. All infections identified in juvenile ducks represented newly acquired infections whereas some proportion of infections in adults were likely chronic. Chronic infections are generally associated with lower parasite loads and adult birds presumably have more developed immune response to parasite infection [1]. We also found support for relationships between body condition and *Leucocytozoon* infection, but the direction of this relationship varied by duck species; northern pintails in poorer condition were more likely to be infected, but we found an opposite effect for American wigeon. In previous experimental studies using wild-strain mallards in captivity, *Leucocytozoon* infection imposed no severe morbidity on ducklings or measureable effects on duckling growth rates [46, 84]. Susceptibility and host immune response to hematozoa is known to vary among avian taxa [9, 33], and experimental studies have shown variation in species-specific tolerance to infections with the same hematozoa lineages [6, 8]. Thus, our results may represent species-specific variation in immune response to *Leucocytozoon* infection. The use of quantitative molecular approaches that determine relative parasite load associated with infections (e.g. [85]) would allow for assessment of variation in body condition relative to parasitemia and may provide improved insight into the epidemiology of hematozoa infections.

## Conclusions

Infections of *Leucocytozoon*, *Haemoproteus*, and *Plasmodium* parasites were common in all species of adult and juvenile ducks sampled, demonstrating an established host-parasite system occurring at the interface of the sub-Arctic and Arctic. Our results corroborate recent evidence in songbirds that *Plasmodium* transmission is established as far north as 65° in Alaska, and extends these findings to multiple waterfowl species. Variation in the prevalence and molecular detection of hematozoa parasites in wild ducks is influenced by parasite genus, seasonal timing, and a number of host traits including species, age, and sex. These factors should be controlled for in future studies to allow for direct comparisons of prevalence and assessment of potential climate-mediated changes in parasite abundance and distribution. Our study provides data on within-host relationships between hematozoa and IAV, showing that infection with these parasitic and viral agents is independent. We observed a strong positive relationship in the occurrence of *Leucocytozoon* and *Haemoproteus* parasites, suggesting that infection by parasites in one or both genera is enhanced by existing infection with the other, or that encounter rates of hosts and genus-specific vectors are correlated. Studies designed to identify arthropod vectors associated with hematozoa transmission in sub-Arctic and Arctic habitats are needed to provide detailed insight into co-infection dynamics.

## Additional files

Additional file 1: This supporting file contains AIC_c_ model selection results for estimating probability of hematozoa infection (Ψ) and probability of detecting hematozoa mtDNA (*p*) for three hematozoa genera (*Leucocytozoon*, *Haemoproteus*, *Plasmodium*) in each of three stages of model selection (Tables S1-S12). Models are ranked by ΔAIC_c_ values. (XLSX 49 kb) Additional file 2: This supporting file contains estimates of *Leucocytozoon* prevalence relative to co-infection with *Haemoproteus* (Table S13), estimated *Haemoproteus* prevalence relative to co-infection with *Leucocytozoon* (Table S14), estimated *Haemoproteus* prevalence relative to body condition (Table S15), and estimated *Leucocytozoon* prevalence relative to body condition (Table S16). (PDF 66 kb)
