# Supplementary material for: Detection, prevalence, and transmission of avian hematozoa in waterfowl at the Arctic/sub-Arctic interface: co-infections, viral interactions, and sources of variation
Source: Parasit Vectors. 2016 Jul 7;9:390. doi: 10.1186/s13071-016-1666-3 (PMC4936110; doi:10.1186/s13071-016-1666-3)
Supplement: Additional file 2: — This supporting file contains estimates of Leucocytozoon prevalence relative to co-infection with Haemoproteus (Table S13), estimated Haemoproteus prevalence relative to co-infection with Leucocytozoon (Table S14), estimated Haemoproteus prevalence relative to body condition (Table S15), and estimated Leucocytozoon prevalence relative to body condition (Table S16). (PDF 66 kb) [file 13071_2016_1666_MOESM2_ESM.pdf]

Table S13. Class-specific estimates of *Leucocytozoon* prevalence ($\hat{\Psi}$) and 95% CIs relative to co-infection status with *Haemoproteus*.

				Without *Haemoproteus*				With *Haemoproteus*				Mean			
**Species**	**Age**	**Sex**	**Month**	$\hat{\Psi}$	**SE**	**LCI**	**UCI**	$\hat{\Psi}$	**SE**	**LCI**	**UCI**	$\hat{\Psi}$	**SE**	**LCI**	**UCI**
Mallard	Ad	M	May	0.40	0.10	0.23	0.60	0.62	0.09	0.43	0.78	0.55	0.10	0.36	0.72
	Ad	F	May	0.14	0.07	0.05	0.33	0.28	0.11	0.11	0.54	0.18	0.08	0.07	0.40
	Ad	B	Jun	0.69	0.10	0.47	0.85	0.85	0.06	0.69	0.93	0.80	0.07	0.62	0.91
	Ad	B	Aug	0.53	0.04	0.45	0.61	0.73	0.04	0.65	0.81	0.60	0.04	0.52	0.68
	Juv	B	Aug	0.11	0.02	0.07	0.15	0.22	0.05	0.15	0.33	0.11	0.02	0.08	0.16
Northern pintail	Ad	M	May	0.44	0.08	0.30	0.59	0.66	0.07	0.51	0.79	0.51	0.08	0.36	0.65
	Ad	F	May	0.16	0.07	0.06	0.35	0.31	0.12	0.13	0.57	0.22	0.09	0.09	0.46
	Ad	B	Jun	0.65	0.08	0.48	0.78	0.82	0.06	0.68	0.90	0.71	0.07	0.55	0.83
	Ad	B	Aug	0.45	0.04	0.37	0.53	0.66	0.05	0.56	0.75	0.50	0.04	0.42	0.58
	Juv	B	Aug	0.38	0.03	0.32	0.45	0.60	0.05	0.50	0.70	0.40	0.03	0.33	0.46
American green-winged teal	Ad	B	Aug	0.55	0.06	0.43	0.66	0.75	0.05	0.63	0.83	0.61	0.06	0.49	0.71
	Juv	B	Aug	0.28	0.04	0.21	0.37	0.49	0.06	0.36	0.61	0.29	0.04	0.21	0.38
American wigeon	Ad	M	May	0.81	0.06	0.68	0.90	0.91	0.03	0.83	0.96	0.86	0.05	0.75	0.93
	Ad	F	May	0.50	0.12	0.28	0.72	0.71	0.10	0.48	0.87	0.53	0.12	0.30	0.74
	Ad	B	Jun	0.93	0.07	0.64	0.99	0.97	0.03	0.81	1.00	0.96	0.04	0.78	1.00
Lesser scaup	Ad	M	May	0.57	0.07	0.43	0.70	0.76	0.06	0.63	0.86	0.63	0.07	0.49	0.75
	Ad	F	May	0.24	0.09	0.11	0.44	0.43	0.12	0.22	0.66	0.28	0.10	0.13	0.49

Estimates of mean prevalence were calculated using class-specific apparent prevalence of *Haemoproeteus*. Age was classified as adult (Ad) or juvenile (Juv); B depicts estimates for both sexes. Month refers to the capture occasions when birds were sampled (May = 17 May – 27 May; Jun = 10 June – 19 June; Aug = 28 July – 20 August).

Table S14. Class-specific estimates of *Haemoproteus* prevalence ($\widehat{\Psi}$) and 95% CIs relative to co-infection status with *Leucocytozoon*.

				Without *Leucocytozoon*				With *Leucocytozoon*				Mean			
**Species**	**Age**	**Sex**	**Month**	$\widehat{\Psi}$	**SE**	**LCI**	**UCI**	$\widehat{\Psi}$	**SE**	**LCI**	**UCI**	$\widehat{\Psi}$	**SE**	**LCI**	**UCI**
Mallard	Ad	M	May	0.56	0.10	0.36	0.74	0.73	0.08	0.55	0.86	0.67	0.09	0.47	0.81
	Ad	F	May	0.45	0.14	0.21	0.71	0.64	0.13	0.36	0.85	0.45	0.14	0.21	0.71
	Ad	B	Jun	0.52	0.11	0.32	0.72	0.71	0.09	0.52	0.85	0.67	0.09	0.48	0.82
	Ad	M	Aug	0.33	0.05	0.23	0.44	0.52	0.06	0.41	0.62	0.45	0.05	0.34	0.55
	Ad	F	Aug	0.16	0.04	0.10	0.24	0.30	0.05	0.21	0.41	0.23	0.04	0.15	0.32
	Juv	M	Aug	0.07	0.03	0.03	0.14	0.13	0.05	0.06	0.26	0.07	0.03	0.03	0.15
Northern pintail	Ad	M	May	0.23	0.06	0.13	0.37	0.39	0.08	0.25	0.56	0.30	0.07	0.18	0.45
	Ad	F	May	0.16	0.08	0.06	0.37	0.29	0.12	0.12	0.57	0.29	0.12	0.12	0.57
	Ad	B	Jun	0.20	0.06	0.11	0.34	0.35	0.08	0.21	0.52	0.30	0.07	0.18	0.46
	Ad	M	Aug	0.04	0.03	0.01	0.13	0.09	0.05	0.03	0.25	0.06	0.03	0.02	0.17
	Ad	F	Aug	0.24	0.04	0.17	0.33	0.41	0.05	0.31	0.51	0.32	0.04	0.24	0.41
	Juv	B	Aug	0.05	0.01	0.03	0.09	0.11	0.03	0.06	0.17	0.07	0.02	0.04	0.11
American green-winged teal	Ad	M	Aug	0.17	0.05	0.09	0.28	0.30	0.07	0.19	0.45	0.23	0.06	0.14	0.37
	Ad	F	Aug	0.23	0.07	0.12	0.41	0.40	0.10	0.24	0.60	0.34	0.09	0.19	0.53
	Juv	B	Aug	0.04	0.02	0.02	0.08	0.08	0.03	0.04	0.17	0.05	0.02	0.02	0.10
American wigeon	Ad	M	May	0.23	0.06	0.14	0.36	0.39	0.07	0.27	0.53	0.36	0.07	0.25	0.50
	Ad	F	May	0.16	0.07	0.07	0.34	0.30	0.11	0.13	0.53	0.23	0.09	0.10	0.45
	Ad	B	Jun	0.63	0.12	0.38	0.83	0.79	0.08	0.59	0.91	0.79	0.08	0.58	0.91
Lesser scaup	Ad	M	May	0.20	0.05	0.12	0.32	0.36	0.07	0.23	0.50	0.30	0.06	0.19	0.43
	Ad	F	May	0.14	0.06	0.06	0.31	0.26	0.10	0.11	0.50	0.16	0.07	0.06	0.34

Estimates of mean prevalence were calculated using class-specific apparent prevalence of *Leucocytozoon*. Age was classified as adult (Ad) or juvenile (Juv); B depicts estimates for both sexes. Month refers to the capture occasions when birds were sampled (May = 17 May – 27 May; Jun = 10 June – 19 June; Aug = 28 July – 20 August).

Table S15. Class-specific estimates of *Leucocytozoon* prevalence ($\hat{\Psi}$) and 95% CIs relative to body condition index (BCI).

				BCI = 10th percentile				BCI = 90th percentile				BCI = 50th percentile			
**Species**	**Age**	**Sex**	**Month**	$\hat{\Psi}$	**SE**	**LCI**	**UCI**	$\hat{\Psi}$	**SE**	**LCI**	**UCI**	$\hat{\Psi}$	**SE**	**LCI**	**UCI**
Mallard	Ad	M	May	0.66	0.09	0.47	0.81	0.64	0.09	0.45	0.80	0.65	0.09	0.46	0.80
	Ad	F	May	0.50	0.14	0.25	0.75	0.47	0.14	0.23	0.73	0.49	0.14	0.24	0.74
	Ad	M	Jun	0.68	0.09	0.49	0.83	0.65	0.10	0.45	0.81	0.67	0.09	0.48	0.82
	Ad	F	Jun	0.67	0.09	0.48	0.82	0.67	0.09	0.47	0.82	0.67	0.09	0.48	0.82
	Ad	M	Aug	0.47	0.06	0.35	0.58	0.43	0.06	0.32	0.55	0.45	0.05	0.35	0.55
	Ad	F	Aug	0.25	0.05	0.17	0.35	0.22	0.05	0.14	0.32	0.23	0.04	0.16	0.32
	**Juv**	**M**	**Aug**	**0.10**	**0.04**	**0.05**	**0.21**	**0.04**	**0.02**	**0.02**	**0.10**	**0.07**	**0.03**	**0.03**	**0.14**
	**Juv**	**F**	**Aug**	**0.11**	**0.04**	**0.05**	**0.22**	**0.05**	**0.02**	**0.02**	**0.11**	**0.07**	**0.03**	**0.03**	**0.14**
Northern pintail	Ad	M	May	0.32	0.07	0.20	0.48	0.30	0.07	0.18	0.45	0.31	0.07	0.19	0.46
	Ad	F	May	N/A				N/A				0.18	0.09	0.07	0.41
	Ad	M	Jun	0.32	0.08	0.19	0.50	0.29	0.08	0.16	0.46	0.31	0.07	0.18	0.47
	Ad	F	Jun	0.33	0.08	0.19	0.50	0.29	0.08	0.16	0.46	0.31	0.07	0.18	0.47
	Ad	M	Aug	0.07	0.04	0.02	0.19	0.06	0.03	0.02	0.17	0.06	0.04	0.02	0.18
	Ad	F	Aug	0.35	0.05	0.25	0.45	0.31	0.05	0.22	0.42	0.33	0.04	0.25	0.42
	**Juv**	**M**	**Aug**	**0.12**	**0.04**	**0.07**	**0.21**	**0.04**	**0.02**	**0.02**	**0.08**	**0.07**	**0.02**	**0.04**	**0.11**
	**Juv**	**F**	**Aug**	**0.11**	**0.03**	**0.06**	**0.18**	**0.04**	**0.02**	**0.02**	**0.09**	**0.07**	**0.02**	**0.04**	**0.11**
American green-winged teal	Ad	M	Aug	0.26	0.06	0.15	0.40	0.23	0.06	0.13	0.36	0.24	0.06	0.15	0.37
	Ad	F	Aug	0.36	0.09	0.20	0.55	0.33	0.09	0.18	0.52	0.34	0.09	0.20	0.53
	**Juv**	**M**	**Aug**	**0.08**	**0.03**	**0.03**	**0.16**	**0.03**	**0.01**	**0.01**	**0.07**	**0.05**	**0.02**	**0.02**	**0.10**
	**Juv**	**F**	**Aug**	**0.08**	**0.03**	**0.04**	**0.18**	**0.03**	**0.01**	**0.01**	**0.07**	**0.05**	**0.02**	**0.02**	**0.10**
American wigeon	Ad	M	May	0.38	0.07	0.26	0.52	0.36	0.07	0.24	0.49	0.37	0.07	0.25	0.50
	Ad	F	May	0.24	0.10	0.10	0.47	0.21	0.09	0.09	0.43	0.23	0.09	0.10	0.44
	Ad	M	Jun	0.79	0.08	0.59	0.91	0.78	0.09	0.56	0.90	0.79	0.08	0.58	0.91
	Ad	F	Jun	0.79	0.08	0.58	0.91	0.78	0.09	0.57	0.91	0.79	0.08	0.58	0.91
Lesser scaup	Ad	M	May	0.31	0.07	0.19	0.46	0.29	0.06	0.18	0.43	0.30	0.06	0.19	0.43
	Ad	F	May	0.18	0.08	0.08	0.38	0.17	0.07	0.07	0.36	0.17	0.07	0.07	0.37

Bold indicates estimates from BCI coefficient with 85% CIs that did not contain zero. Age was classified as adult (Ad) or juvenile (Juv). Month refers to the capture occasions when birds were sampled (May = 17 May – 27 May; Jun = 10 June – 19 June; Aug = 28 July – 20 August). N/A indicates insufficient sample size to estimate BCI effect.

Table S16. Class-specific estimates of *Haemoproteus* prevalence ($\widehat{\Psi}$) and 95% CIs relative to body condition index (BCI).

				BCI = 10th percentile				BCI = 90th percentile				BCI = 50th percentile			
Species	Age	Sex	Month	$\widehat{\Psi}$	SE	LCI	UCI	$\widehat{\Psi}$	SE	LCI	UCI	$\widehat{\Psi}$	SE	LCI	UCI
Mallard	Ad	M	May	0.52	0.10	0.34	0.70	0.56	0.10	0.37	0.74	0.54	0.09	0.36	0.71
	Ad	F	May	0.19	0.09	0.07	0.43	0.22	0.10	0.08	0.46	0.21	0.09	0.08	0.44
	Ad	M	Jun	0.78	0.08	0.58	0.90	0.81	0.07	0.63	0.92	0.79	0.08	0.61	0.90
	Ad	F	Jun	0.79	0.08	0.60	0.90	0.80	0.07	0.62	0.91	0.79	0.08	0.61	0.90
	Ad	M	Aug	0.57	0.05	0.46	0.67	0.62	0.05	0.52	0.72	0.60	0.04	0.52	0.67
	Ad	F	Aug	0.56	0.06	0.45	0.67	0.63	0.05	0.52	0.73	0.60	0.04	0.52	0.67
	Juv	M	Aug	0.10	0.02	0.06	0.15	0.12	0.03	0.08	0.19	0.11	0.02	0.07	0.16
	Juv	F	Aug	0.10	0.02	0.06	0.16	0.12	0.03	0.08	0.18	0.11	0.02	0.07	0.16
Northern pintail	**Ad**	**M**	**May**	**0.57**	**0.08**	**0.42**	**0.71**	**0.44**	**0.08**	**0.30**	**0.59**	**0.51**	**0.07**	**0.37**	**0.65**
	**Ad**	**F**	**May**	**N/A**				**N/A**				**0.19**	**0.08**	**0.07**	**0.40**
	**Ad**	**M**	**Jun**	**0.77**	**0.07**	**0.62**	**0.88**	**0.61**	**0.09**	**0.43**	**0.77**	**0.70**	**0.07**	**0.55**	**0.83**
	**Ad**	**F**	**Jun**	**0.78**	**0.07**	**0.63**	**0.88**	**0.62**	**0.09**	**0.44**	**0.77**	**0.70**	**0.07**	**0.55**	**0.83**
	**Ad**	**M**	**Aug**	**0.57**	**0.05**	**0.48**	**0.66**	**0.41**	**0.05**	**0.32**	**0.51**	**0.50**	**0.04**	**0.42**	**0.58**
	**Ad**	**F**	**Aug**	**0.58**	**0.05**	**0.48**	**0.67**	**0.40**	**0.05**	**0.30**	**0.51**	**0.50**	**0.04**	**0.42**	**0.58**
	**Juv**	**M**	**Aug**	**0.50**	**0.05**	**0.40**	**0.59**	**0.31**	**0.04**	**0.23**	**0.40**	**0.40**	**0.03**	**0.33**	**0.46**
	**Juv**	**F**	**Aug**	**0.48**	**0.04**	**0.39**	**0.56**	**0.32**	**0.04**	**0.25**	**0.40**	**0.40**	**0.03**	**0.33**	**0.46**
American green-winged teal	Ad	M	Aug	0.62	0.07	0.47	0.75	0.58	0.07	0.43	0.72	0.60	0.06	0.48	0.70
	Ad	F	Aug	0.61	0.07	0.48	0.74	0.58	0.07	0.45	0.71	0.60	0.06	0.48	0.70
	Juv	M	Aug	0.31	0.06	0.20	0.44	0.27	0.06	0.18	0.40	0.29	0.04	0.22	0.38
	Juv	F	Aug	0.31	0.07	0.20	0.46	0.27	0.05	0.18	0.39	0.29	0.04	0.22	0.38
American wigeon	**Ad**	**M**	**May**	**0.71**	**0.09**	**0.50**	**0.86**	**0.95**	**0.03**	**0.82**	**0.99**	**0.88**	**0.05**	**0.75**	**0.94**
	**Ad**	**F**	**May**	**0.25**	**0.13**	**0.08**	**0.57**	**0.87**	**0.11**	**0.48**	**0.98**	**0.61**	**0.13**	**0.34**	**0.83**
	**Ad**	**M**	**Jun**	**0.93**	**0.07**	**0.63**	**0.99**	**0.99**	**0.02**	**0.88**	**1.00**	**0.97**	**0.03**	**0.80**	**1.00**
	**Ad**	**F**	**Jun**	**0.94**	**0.06**	**0.67**	**0.99**	**0.98**	**0.03**	**0.83**	**1.00**	**0.97**	**0.03**	**0.80**	**1.00**
Lesser scaup	Ad	M	May	0.66	0.11	0.42	0.83	0.60	0.10	0.41	0.77	0.63	0.07	0.49	0.75
	Ad	F	May	0.29	0.13	0.11	0.58	0.25	0.11	0.10	0.51	0.27	0.10	0.13	0.49

Bold indicates estimates from BCI coefficient with 85% CIs that did not contain zero. Age was classified as adult (Ad) or juvenile (Juv). Month refers to the capture occasions when birds were sampled (May = 17 May – 27 May; Jun = 10 June – 19 June; Aug = 28 July – 20 August). N/A indicates insufficient sample size to estimate BCI effect.
